# Estrogen influences class-switched memory B cell frequency only in humans with two X chromosomes

**DOI:** 10.1084/jem.20241253

**Published:** 2025-03-06

**Authors:** Hannah Peckham, Anna Radziszewska, Justyna Sikora, Nina M. de Gruijter, Restuadi Restuadi, Melissa Kartawinata, Lucia Martin-Gutierrez, George A. Robinson, Claire T. Deakin, Lucy R. Wedderburn, Elizabeth C. Jury, Gary Butler, Emma S. Chambers, Elizabeth C. Rosser, Coziana Ciurtin

**Affiliations:** 1 Centre for Adolescent Rheumatology Versus Arthritis at UCL, UCLH and GOSH, London, UK; 2 Centre for Rheumatology, University College London, London, UK; 3 Infection, Immunity and Inflammation Research and Teaching Department – UCL Great Ormond Street Institute of Child Health, London, UK; 4 University College London Hospital, London, UK; 5 NIHR Biomedical Research Centre at Great Ormond Street Hospital, London, UK; 6 School of Population Health, Faculty of Medicine and Health, University of New South Wales, Sydney, Australia; 7 Centre for Immunobiology, Blizard Institute, Queen Mary University of London, London, UK

## Abstract

Sex differences in immunity are well-documented, though mechanisms underpinning these differences remain ill-defined. Here, in a human-only ex vivo study, we demonstrate that postpubertal cisgender females have higher levels of CD19^+^CD27^+^IgD^−^ class-switched memory B cells compared with age-matched cisgender males. This increase is only observed after puberty and before menopause, suggesting a strong influence for sex hormones. Accordingly, B cells express high levels of estrogen receptor 2 (ESR2), and class-switch–regulating genes are enriched for ESR2-binding sites. In a gender-diverse cohort, blockade of natal estrogen in transgender males (XX karyotype) reduced class-switched memory B cell frequency, while gender-affirming estradiol treatment in transgender females (XY karyotype) did not increase these levels. In postmenopausal cis-females, class-switched memory B cells were increased in those taking hormone replacement therapy (HRT) compared with those who were not. These data demonstrate that sex hormones and chromosomes work in tandem to impact immune responses, with estrogen only influencing the frequency of class-switched memory B cells in individuals with an XX chromosomal background.

## Teaser

Sex hormones and chromosomes work in tandem to impact immune responses, with estrogen influencing class-switched memory B cell frequency exclusively in individuals with an XX chromosomal background.

## Graphical Abstract

**Figure:**
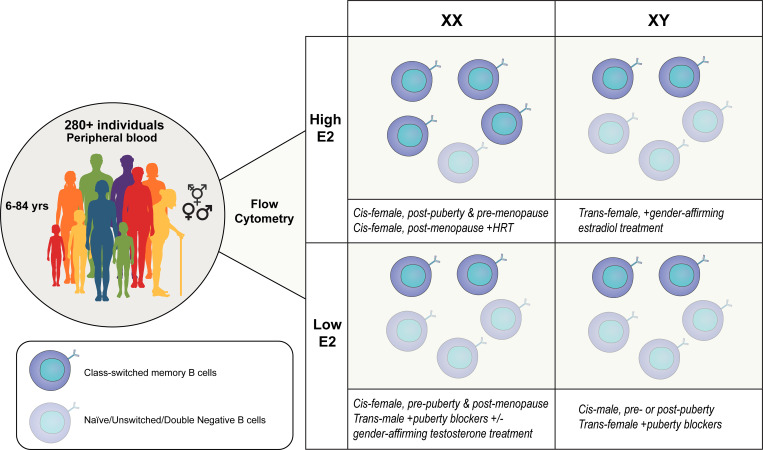

## Introduction

The presence of a sex bias in the human immune system has been widely reported in recent decades. Studies from across the field of infection and immunity highlight the need to disaggregate research findings by sex and gender. Typically, cisgender males (cis-males) appear more at risk of severe outcomes from many infectious diseases, as evidenced by the recent coronavirus disease 2019 (COVID-19) pandemic (Peckham et al., 2020; Biswas et al., 2020; Klein et al., 2020; Scully et al., 2020; Takahashi and Iwasaki, 2021), while cisgender females (cis-females) are more likely to develop humoral autoimmune diseases such as systemic lupus erythematosus (SLE), Sjögren’s disease, dermatomyositis, and systemic sclerosis (Brinks et al., 2016; Lim et al., 2014; Izmirly et al., 2017; Conrad et al., 2023; Beydon et al., 2024; Phillippi et al., 2017; Kronzer et al., 2023; Ciaffi et al., 2021; Hughes et al., 2020; Zhong et al., 2019). Very little is known regarding immune-mediated disease outcomes in transgender females and males (trans-females and -males) or nonbinary people (those identifying neither as exclusively male nor exclusively female) in the context of gender-affirming hormone therapy (GAHT) treatment (Peckham et al., 2022). There are also relatively few studies investigating how hormonal changes over the full age spectrum influence sex differences in immune responses. Studies that aim to investigate the immunological underpinnings of sex and gender biases across life course in inflammatory disease remain of critical importance.

Socioeconomic determinants relating to differing gender roles are known to play a part in disease risk and outcomes (Bischof et al., 2020; Morgan and Klein, 2019; Bertrand et al., 2024). However, there is strong evidence that sex chromosomes (X and Y) and sex hormones are the primary driving force behind sex biases in immune-mediated conditions. The X chromosome, of which cis-females and trans-males have two copies, encodes the most immune-relevant genes from the human genome (Bianchi et al., 2012). X chromosome inactivation (XCI) silences the expression of one copy of each X gene (in people with two X chromosomes), but a subset of genes may exhibit variable tissue-specific differences in XCI. Key examples include immune-related genes such as *TLR7*, which is well-documented in its relevance for immune signalling and implications for autoimmunity (Qu et al., 2015; Wang et al., 2016; Tukiainen et al., 2017; Souyris et al., 2018; Oghumu et al., 2019); CD40 ligand (*CD40-L*), vital to T and B cell activation signalling that maintains immune tolerance (Pucino et al., 2020); NF-κB, which is involved in B cell fate decisions throughout the immune response (Guldenpfennig et al., 2023); the IL-2 receptor gamma chain (*IL-2RG*), mutations of which lead to X-linked severe combined immunodeficiency (SCID) (Tuovinen et al., 2020); and Bruton’s tyrosine kinase (Hagen et al., 2020), which regulates B cell activation and differentiation. XCI is triggered on the future inactive X by the buildup of X-inactive specific transcript (*XIST*) long coding RNA (Loda et al., 2022), which recruits a program of gene silencing factors. By perturbing the expression of *Xist* in a mouse model, a recent study proposed a direct link between dysregulated XCI and the development of autoimmunity (Huret et al., 2024). Furthermore, males with Klinefelter syndrome, who have one or more additional X chromosomes, have an increased risk of developing autoimmune disorders such as SLE, systemic sclerosis, and Sjögren’s disease (Dillon et al., 2011; Scofield et al., 2008; Rovenský et al., 2014; Harris et al., 2016), despite the presence of male sex hormones.

However, sex biases in immune-mediated diseases are not consistent throughout age. Disorders such as asthma and atopy, characterized by male preponderance before puberty, have increased female prevalence during reproductive years (Osman, 2003). In addition, the onset of various adolescent autoimmune conditions at the time of puberty (Massias et al., 2020; Whincup et al., 2001; Cattalini et al., 2019) suggests that increased endogenous exposure to sex hormones, as well as age, has a direct impact on immune system function (Nikolich-Žugich, 2017; Sadighi Akha, 2018). This is further supported by the changes in immune-mediated disease risk and severity following the decline of sex hormone levels associated with menopause (Gubbels Bupp, 2015; Desai and Brinton, 2019; Costeira et al., 2021) and the potential for hormone replacement therapy (HRT; sometimes referred to as menopausal hormone therapy) to improve COVID-19 outcomes in menopausal women (Seeland et al., 2020; Dambha-Miller et al., 2022). There is also a growing body of evidence to suggest that sex hormones influence the development of autoimmunity (McMurray and May, 2003; Petri, 2008; Kim et al., 2022), allergy, and asthma (McCleary et al., 2018; Han et al., 2020) and the effectiveness of pathogen clearance (Ruggieri et al., 2018; vom Steeg and Klein, 2019; Gupta et al., 2022). The principal sex hormones in humans are testosterone (an androgen), progesterone (“P4,” a progestin), and the four major forms of estrogen (estrone/estradiol/estriol/estetrol, or E1/2/3/4). Intracellular receptors for these hormones are found on numerous cell types, including many immune cells (Uhlen et al., 2019). For estrogen, the main receptors in humans are estrogen receptor α (ERα*/ESR1*) and estrogen receptor β (ERβ*/ESR2*), while the androgen receptor (*AR*) is the main receptor for testosterone. These receptors act as key transcriptional regulators throughout the body, with ligation by their cognate hormone impacting both physiological, metabolic, and immune system function (Hayakawa et al., 2022; Hoffmann et al., 2023; Lucas-Herald and Touyz, 2022; Gegenhuber et al., 2022).

Studies comparing the immune system between females and males have long established that there are sex-based differences in B cell responses, with females displaying higher basal and postvaccination antibody titers than males (Flanagan et al., 2017). Studies in humans have suggested that this is due to the suppressive effect of testosterone (Furman et al., 2014). However, murine literature suggests that estrogen plays a dominant role in regulating B cell survival (Bynoe et al., 2000; Grimaldi et al., 2002) and class-switch recombination (CSR) (Mai et al., 2010; Pauklin et al., 2009; White et al., 2011; Jones et al., 2016, 2019), the process by which B cell receptors switch the constant region of their immunoglobulin from initial IgM and IgD isotypes to IgG, IgA, or IgE. IgG antibodies can pass from the blood into tissue sites, and thus have particular importance in viral clearance and immunization response. In the context of autoimmunity, IgG antibodies also make up the bulk of the pathogenic autoantibodies (Olsen and Karp, 2014; Lin and Li, 2009; Villalta et al., 2013; Jayakanthan et al., 2016), being found in the kidney biopsies of patients with SLE nephritis (Bijl et al., 2002; Kenderov et al., 2002) and demonstrated as damage-causing in mouse models (Madaio et al., 1987; Pankewycz et al., 1987). Thus, heightened IgG production may also contribute to the increased risk of autoimmune development in females. Conversely, autoantibodies of the IgG subclass IgG4 have been shown to have protective or anti-inflammatory effects (Jiang et al., 2022; Pan et al., 2020). The molecular mechanism, as well as the relative contribution of sex hormones and sex chromosomes, to differences in B cell class switching and class-switched memory B cell frequency in humans remains ill-defined.

Here, we have utilized a unique age- and gender-diverse cohort of prepubertal, postpubertal, and postmenopausal cis-females, age-matched cis-males, and postpubertal transgender healthy young people to investigate the relative contribution of sex hormones and sex chromosomes in driving differences within the immune, and more specifically, humoral immune system across the life course. Collectively, our data demonstrate that female sex chromosomes and sex hormones work in tandem to influence the frequency of CD19^+^CD27^+^IgD^−^ class-switched memory B cells in humans across the life course. Due to the important role that B cells have in controlling numerous immune-mediated disease outcomes, these data provide significant insights into many clinically observed sex biases.

## Results and discussion

### Postpubertal cis-females have an increase in class-switched memory B cells within peripheral blood when compared with postpubertal cis-males

**Figure 1.**
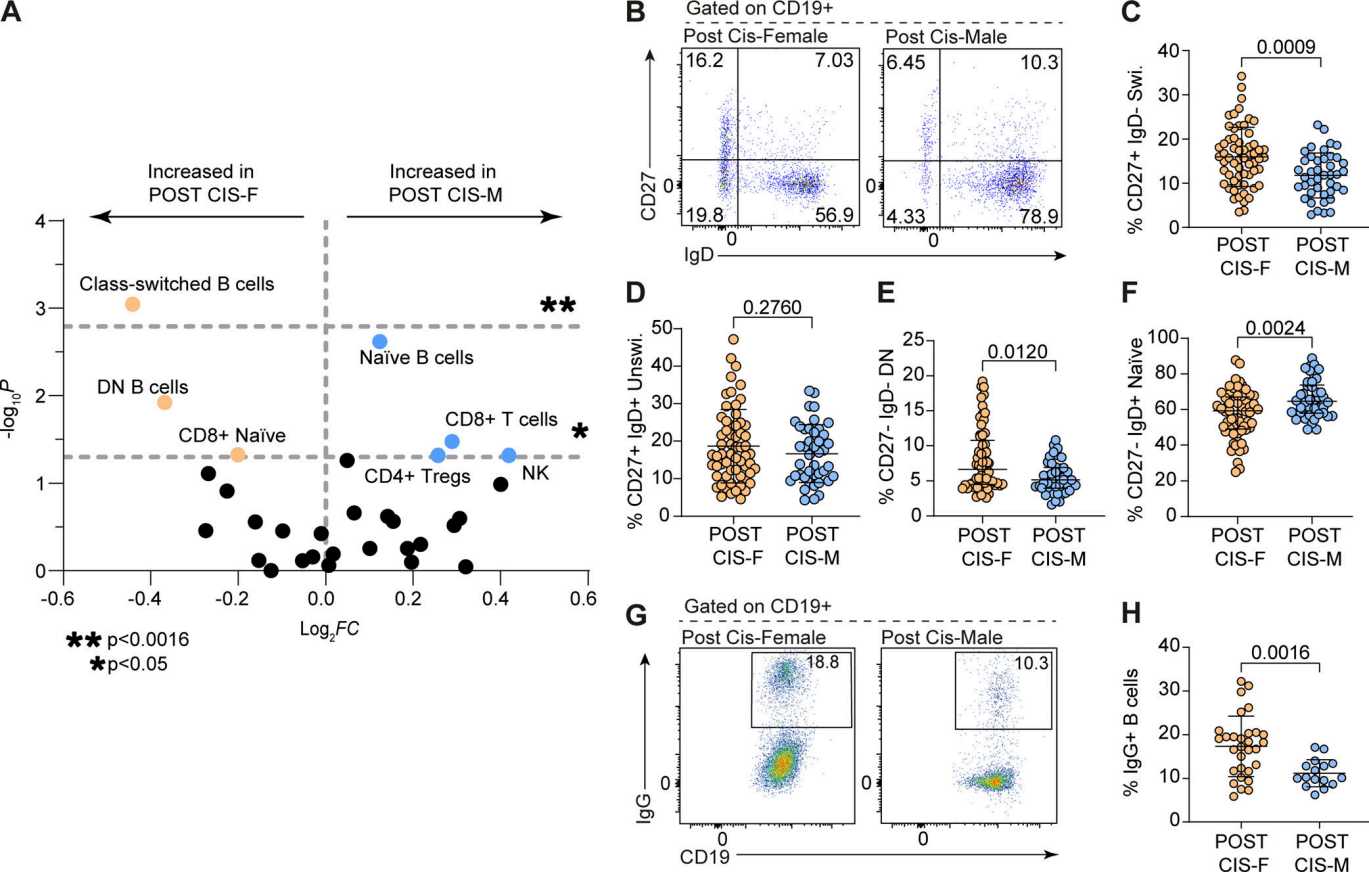
**Healthy postpubertal cis-females have a higher percentage of class-switched memory B cells than age-matched healthy cis-males. (A)** Volcano plot showing −log_10_ P values and log_2_ fold changes of flow cytometric relative percentages of peripheral blood immune cell subtypes between postpubertal cis-female (POST CIS-F) and postpubertal cis-male (POST CIS-M) healthy donors (horizontal dashed lines show P < 0.05; denoted by * and multiple testing adjusted significance level of P < 0.0016; denoted by **). **(B–F)** Representative flow cytometry plots and summary dot plots comparing postpubertal cis-female and postpubertal cis-male percentages of (C) class-switched memory (CD27^+^ IgD^−^; P = 0.0009), (D) unswitched (CD27^+^ IgD^+^; P = 0.2760), (E) DN (CD27^−^ IgD^−^; P = 0.0120), and (F) naïve (CD27^−^ IgD^+^; P = 0.0024) B cells (POST CIS-F *n* = 61; POST CIS-M *n* = 42). **(G and H)** Representative flow cytometry plots and summary dot plots comparing postpubertal cis-female and postpubertal cis-male percentages of IgG^+^ B cells (POST CIS-F *n* = 30; POST CIS-M *n* = 17; P = 0.0016). Note: Representative plots shown in G are duplicated in Fig. S2, B and D for clarity of comparison. Unpaired *t* test with mean + SD (C, D, and H) or Mann–Whitney U test with median + IQR (E and F) as appropriate to distribution of data. Swi., class-switched memory; Unswi., unswitched; and Tregs, T regulatory cells.

**Figure S1.**
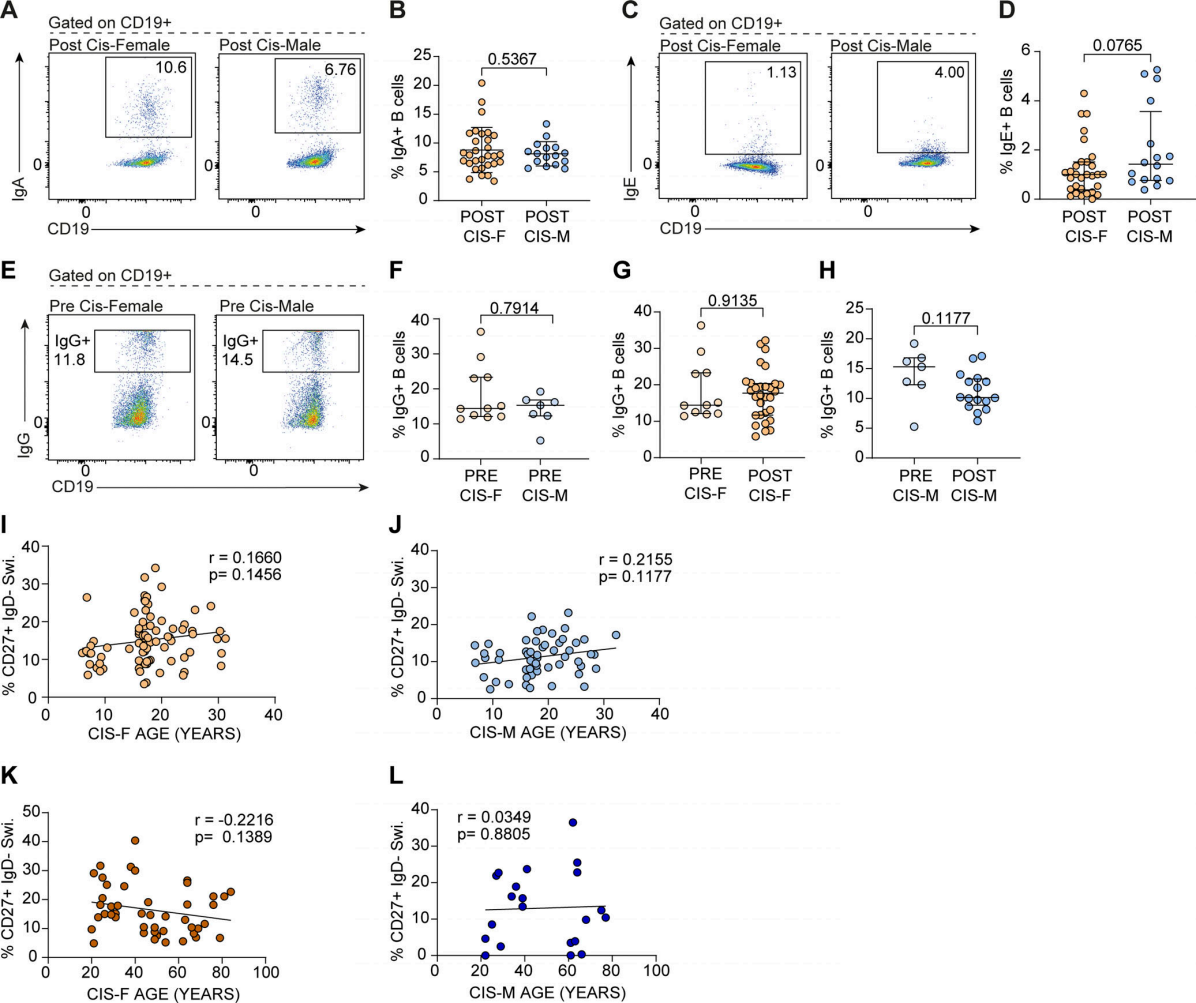
**The frequency of class-switched memory B cells is dependent on puberty and menopause in cis-females. (A and B)** Representative flow cytometry plots (A) and scatter plot (B) showing relative percentages of IgA^+^ B cells in postpubertal cis-females versus postpubertal cis-males (POST CIS-F *n* = 30; POST CIS-M *n* = 17; P = 0.5367). **(C and D)** Representative flow cytometry plots (C) and scatter plot (D) showing relative percentages of IgE^+^ B cells in postpubertal cis-females versus postpubertal cis-males (POST CIS-F *n* = 30; POST CIS-M *n* = 17; P = 0.0765). **(E and F)** Representative flow cytometry plots (E) and scatter plot (F) comparing proportions of IgG^+^ B cells between prepubertal cis-females and prepubertal cis-males (PRE CIS-F *n* = 11; PRE CIS-M *n* = 7; P = 0.7194). **(G and H)** Scatter plots comparing proportions of IgG^+^ B cells between (G) prepubertal cis-females (*n* = 11) and postpubertal cis-females (*n* = 30; P = 0.9135) and (H) prepubertal cis-males (*n* = 7) and postpubertal cis-males (*n* = 17; P = 0.1177). **(I–K)** Pearson test for correlation between age (years) and percentage of IgD^−^ CD27^+^ switched B cells in (I) postpubertal cis-females (*n* = 78; r = 0.1660; P = 0.1456) and (J) postpubertal cis-males (*n* = 54; r = 0.2155; P = 0.1177) in the original PBMC cohort and (K) postpubertal cis-females (*n* = 46; r = −0.2216; P = 0.1389) and (L) postpubertal cis-males (*n* = 21; r = 0.0349; P = 0.9905) in the validation whole blood cohort. Unpaired *t* test with mean + SD (B) or Mann–Whitney U test with median + IQR (D and F–H) as appropriate to distribution of data.

To interrogate which cell population(s) may be behind the observed sex differences in immunity, the proportion of 31 innate and adaptive immune cell types within peripheral blood samples was compared between 103 postpubertal healthy cis-females (POST CIS-F in figures) and cis-males (POST CIS-M in figures) using flow cytometry (Fig. 1 A and Table 1 for demographic information). Multiple immune cell frequencies were differentially expressed based on sex, including natural killer cells, CD4^+^ T regulatory cells, total CD8^+^ T cells, naïve CD8^+^ T cells, naïve (CD27^−^IgD^+^) B cells, and “double negative” (DN CD27^−^IgD^−^) B cells (Fig. 1, A–F). However, CD19^+^CD27^+^IgD^−^ class-switched memory B cells displayed by far the most striking sex difference and were the only population to meet the significance threshold for multiple testing (Fig. 1, A–F). More specifically, cis-females had significantly elevated relative proportions of CD19^+^CD27^+^IgD^−^ class-switched memory B cells compared with cis-males (Fig. 1 C). This increase in CD19^+^CD27^+^IgD^−^ class-switched memory B cells was specifically associated with an increase in IgG^+^ B cells in cis-females compared with cis-males with no significant differences in the levels of IgA^+^ or IgE^+^ B cells between the sexes (Fig. 1, G and H; and Fig. S1, A–D), corroborating previous studies that have shown increased IgG titers in females following vaccination (Fink and Klein, 2018; Fink et al., 2018).

**Table 1.** Demographic information for cisgender (prepubertal and postpubertal) and transgender healthy control sample donors used for the PBMC and whole blood immunophenotyping cohorts

PBMC cohort	Prepubertal cis HC	Postpubertal cis HC	Trans HC	P value; (a) prepubertal vs. postpubertal, (b) postpubertal vs. trans
Total, *n*	26	103	80	-
Gender identity, F:M	15:11	61:42	33:47	-
Mean age, years (range)	8.5 (6–11)	19.9 (14–32)	18.0 (15–19)	(a) N/A (b) 0.0587
Tanner stage 1 (prepuberty) (%)	26 (100)	0 (0)	0 (0)	-
Tanner stage 2–3 (in puberty) (%)	0 (0)	0 (0)	2 (2.5)	-
Tanner stage 4–5 (completing/completed puberty) (%)	0 (0)	103 (100)	78 (97.5)	-
Mean age of menarche, (years) in XX individuals (range)	N/A	12.5 (9–17) NB data on 58/61 donors	12.1 (9–14)	(a) N/A (b) 0.2275
**Ethnicity (%)**				
Asian (any)	2 (7.7)	27 (26.2)	1 (1.3)	(a) 0.0632 (b) <0.0001
Black (any)	0 (0)	9 (8.7)	1 (1.3)	(a) 0.2027 (b) 0.0445
Other/mixed	2 (7.7)	8 (7.8)	6 (7.5)	(a) >0.9999 (b) >0.9999
White (any)	22 (84.6)	59 (57.3)	72 (90)	(a) 0.0119 (b) <0.0001
**Medications**				
Oral contraceptive (%)	0 (0)	10 (9.7)	0 (0)	-
GnRHa “blockers” F:M (%)	N/A	N/A	33:40 (91.3)	-
Estradiol (trans-female; oral/patch) (%)	N/A	N/A	20 (25)	-
Testosterone (trans-male; IM) (%)	N/A	N/A	25 (31.3)	-
Mean months on estradiol (trans-female) (range)	N/A	N/A	13.3 (4.5–23)	-
Mean months on testosterone (trans-male) (range)	N/A	N/A	14.6 (4–43)	
**Whole blood cohort**	**20–40 years**	**40–60 years**	**60+ years**	**P value** **20–40 ** **vs.** ** 60+**
Total, *n*	30	19	25	-
Sex, F:M	19:11	19:0	15:10	>0.9999
Mean age, years (range)	29.4 (21–40)	50.6 (43–58)	68.6 (61–84)	-
Asian (%)	6 (20)	2 (10.5)	4 (16)	>0.9999
Black (%)	0 (0)	1 (5.3)	1 (4)	0.4545
Other/mixed (%)	2 (6.6)	0 (0)	0 (0)	0.4949
Unknown ethnicity (%)	3 (10)	0 (0)	0 (0)	0.2424
White (%)	19 (63.3)	16 (84.2)	20 (80)	0.1569
HRT usage (%)	0 (0)	7 (36.8)	0 (0)	-

HC, healthy control; IM, intramuscular. Fisher’s exact test used to calculate differences between groups.

### Sex differences in the B cell transcriptome between healthy, postpubertal cis-females and cis-males are driven by sex chromosome–encoded genes

**Figure 2.**
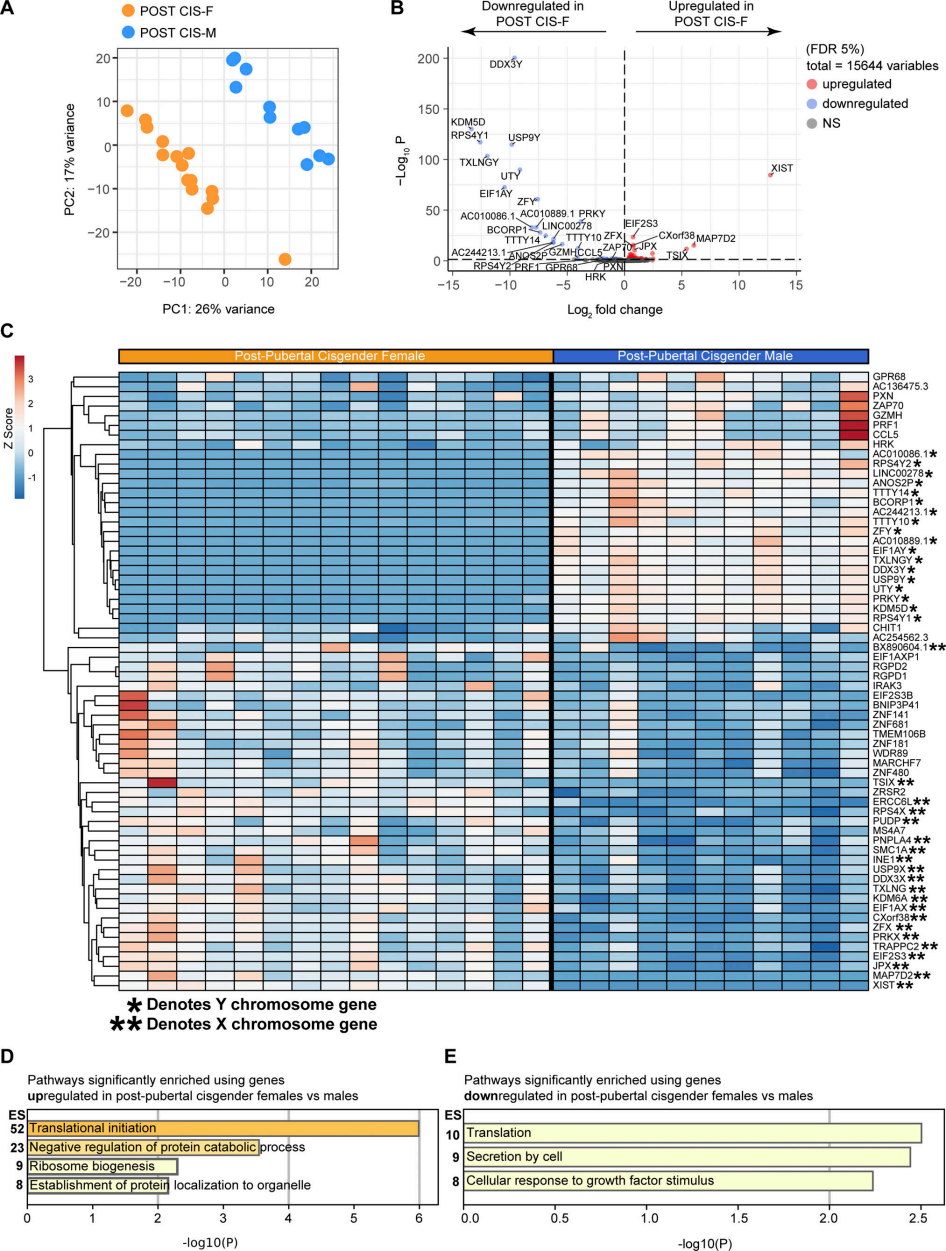
**Sex chromosome genes dominate the differential gene expression between total CD19** ^**+**^ **B cell RNA from healthy, young cis-females and cis-males. (A)** PC analysis plot showing clustering of postpubertal cis-female (POST CIS-F) versus postpubertal cis-male (POST CIS-M) CD19^+^ B cell gene expression. **(B)** Volcano plot shows DEGs that are up- (red) or down- (blue) regulated in postpubertal cis-females compared with postpubertal cis-males. Only those with a Padj <0.05 are labelled as significant. **(C)** Heatmap demonstrating clustering by DEG profiles of postpubertal cis-females versus postpubertal cis-males. **(D and E)** Metascape pathway enrichment analysis shows pathways significantly enriched in analysis of (D) upregulated DEGs and (E) downregulated DEGs in postpubertal cis-females compared with postpubertal cis-males (*n* = 15 POST CIS-F, 11 POST CIS-M). ES, enrichment score (the ratio of the proportion of genes in the list that were associated with the enriched term to the proportion of genes in the genome that are associated with the enriched term). FDR, false discovery rate.

To understand the potential mechanistic underpinnings of the expansion of CD19^+^CD27^+^IgD^−^ class-switched memory B cells in cis-females compared with cis-males, we next analyzed the transcriptome of total CD19^+^ B cells from postpubertal cis-females and postpubertal cis-males via RNA sequencing (RNAseq). The transcriptome of B cells was clearly segregated by sex (Fig. 2 A) with 64 differentially expressed genes (DEGs; full gene list with chromosome number/letter shown in Table S1) between these two groups, of which 28 were downregulated in cis-females compared with cis-males, and 36 were upregulated. 40 (62.5%) of the identified DEGs were found to be encoded on either the X (22/40) or Y (18/40) chromosome (Fig. 2, B and C; and Table S1). As expected, this included genes such as *XIST* and *TSIX*, long noncoding RNA that control silencing of the X chromosomes. The level of expression of *XIST* and *TSIX* varied among cis-females, which could have implications regarding differences in the extent of XCI amongst individuals. In terms of B cell biology, dysregulation in *XIST* has been recently shown to lead to the expansion of the CD11c^+^ atypical B cells in female patients with SLE or COVID-19 (Yu et al., 2021). However, we did not find any correlation between the levels of class-switched memory B cells and *XIST* transcript levels in our cohort (data not shown). Of the DEGs that were not X or Y genes, few had clear known immune functions in B cells, with the exception of *HRK* and *CCL5*, which were downregulated in cis-females, and *IRAK3*, which was upregulated in cis-females. Importantly, *HRK* is in the BCL2 family of proteins, which regulates cell death (Inohara et al., 1997; Spetz et al., 2019; Kaya-Aksoy et al., 2019); chemokine ligand 5 (*CCL5*) is a known chemoattractant (Gauthier et al., 2023; Aldinucci et al., 2020); and IL-1 receptor-associated kinase 3 (*IRAK3*) is a negative regulator of TLR signalling (Pereira and Gazzinelli, 2023). Further studies are needed to interrogate the direct impact of these genes on B cell function and therefore any of our observed sex differences in the B cell compartment. Pathway analysis of DEGs upregulated in cis-females compared with cis-males revealed significant enrichment of gene ontology biological processes (GO BP) pathways entitled translational initiation, negative regulation of protein catabolic process, ribosome biogenesis, and establishment of protein localization to organelle, whilst pathway analysis of DEGs downregulated in cis-females compared with cis-males revealed significant enrichment of GO BP pathways entitled translation, secretion by cell, and cellular response to growth factor stimulation (Fig. 2, D and E). Changes in these pathways further support a role for sexually divergent B cell activation, differentiation, and potentially class-switching profiles, as B cell maturation is well-known to be associated with drastic alterations in cellular structure and translational machinery needed to produce vast amounts of protein in the form of immunoglobulin (Nutt et al., 2015).

### Sex differences in class-switched memory B cell frequencies are not observed between prepubertal cis-females and cis-males

**Figure 3.**
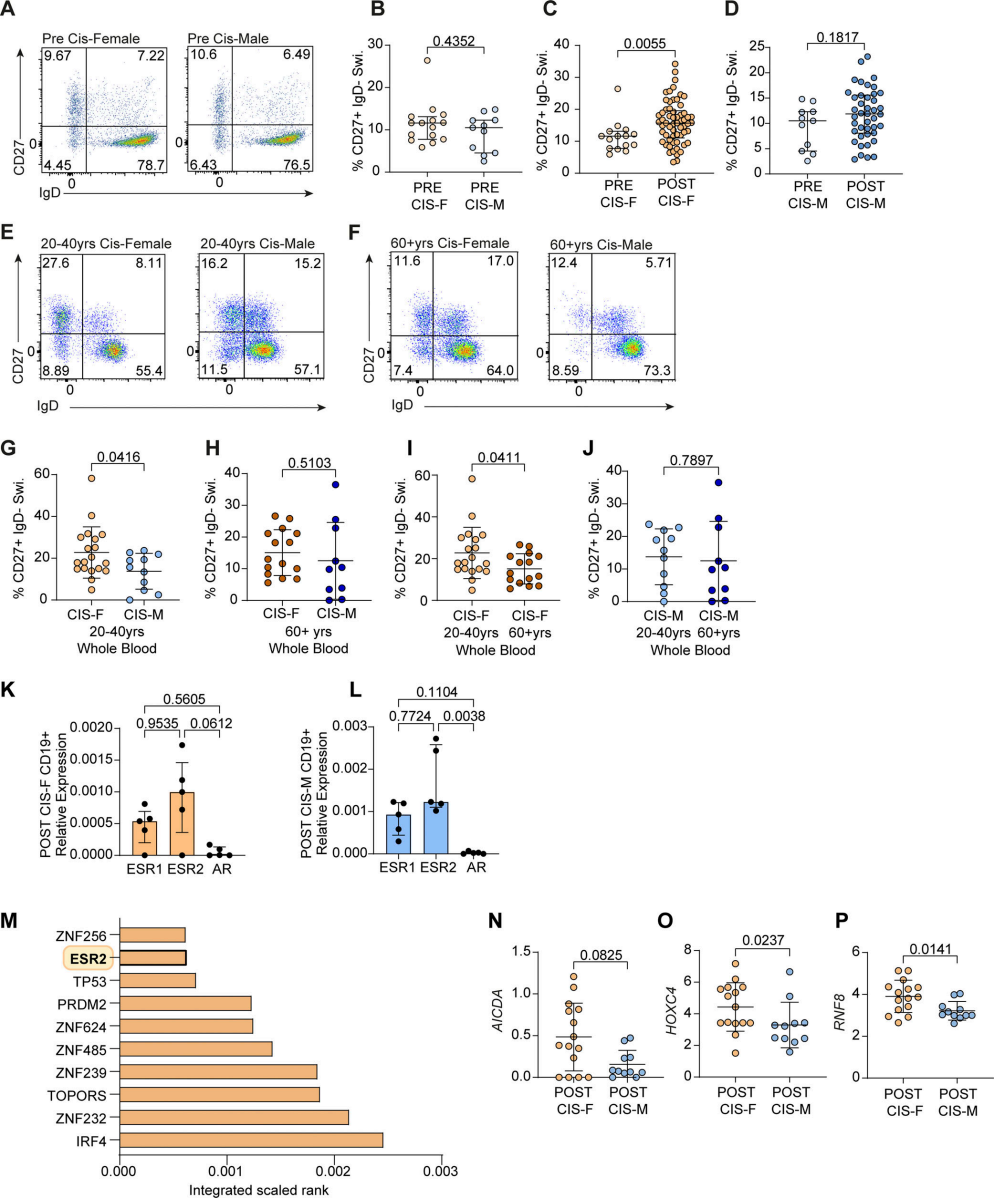
**Estrogen levels across the lifespan are associated with differences in the frequency of class-switched memory B cells in healthy cis-females compared with age-matched males. (A and B)** Representative flow cytometry plots (A) and summary dot plot (B) comparing healthy, prepubertal cis-female (PRE CIS-F *n* = 15) and prepubertal cis-male (PRE CIS-M *n* = 11) relative percentages of class-switched memory (Swi.; CD27^+^ IgD^−^; P = 0.4352). **(C and D)** Comparison of class-switched memory B cell percentages in prepubertal versus postpubertal (C) cis-females (PRE CIS-F *n* = 15; POST CIS-F *n* = 61; P = 0.0055) and (D) cis-males (PRE CIS-M *n* = 11; POST CIS-M *n* = 42; P = 0.1817). **(E and G)** Representative flow cytometry plots (E) and summary dot plot (G) comparing healthy, pre-menopausal 20–40-year-old cis-female (CIS-F 20–40 years; *n* = 19) and age-matched male (CIS-M 20–40 years; *n* = 11) relative percentages of class-switched memory (Swi.; CD27^+^ IgD^−^; P = 0.0416) B cells in whole blood. **(F and H)** Representative flow cytometry plots (F) and summary dot plot (H) comparing healthy, postmenopausal 60+-year-old cis-female (CIS-F 60+ years; *n* = 15) and age-matched male (CIS-M 60+ years; *n* = 10) relative percentages of class-switched memory (Swi.; CD27^+^ IgD^−^; P = 0.5103) B cells in whole blood. **(I and J)** Summary dot plots comparing relative proportions of class-switched memory B cells in whole blood between (I) cis-females, pre- versus postmenopause (P = 0.0411) and (J) 20–40-year old versus 60+-year-old cis-males (P = 0.7897). **(K and L)** Expression of hormone receptors in isolated CD19^+^ cells from postpubertal cis-female (POST CIS-F *n* = 5) and postpubertal cis-male (POST CIS-M *n* = 5) healthy donors, relative to expression of housekeeping gene *RPLP0*, measured by qPCR (ERα, *ESR1*; ERβ, *ESR2*; and *AR*. POST CIS-F: *ESR1* vs. *ESR2* P = 0.9535; *ESR1* vs. *AR *P = 0.5605; and *ESR2* vs. *AR* P = 0.0612; POST CIS-M: *ESR1* vs. *ESR2* P = 0.7724; *ESR1* vs. *AR* P = 0.1104; and *ESR2* vs. *AR* P = 0.0038). **(M)** Top 10 TFs whose putative transcriptional targets are most similar to identified class-switching gene set. Integrated score takes into account results from all ChEA3 TF target libraries, with lower scores indicating higher relevance to the TF. **(N–P)** Comparison of gene counts for key class-switching genes: *AICDA* (P = 0.0825), *HOXC4* (P = 0.0237), and *RNF8* (P = 0.0141) from healthy postpubertal cis-female versus postpubertal cis-male B cell RNAseq. Unpaired *t* test (G–J) with mean + SD shown or Mann–Whitney U test (B–G and M–O) or Kruskal–Wallis test with Dunn’s test (K and L) with median + IQR shown.

If the sex chromosomes are, indeed, solely responsible for the observed sex differences in the frequency of CD19^+^CD27^+^IgD^−^ class-switched memory B cells, it may be expected that these differences would be observed across all age brackets despite changing levels of sex hormones. However, when comparing the frequency of class-switched memory B cells in healthy, prepubertal cis-females (referred to as PRE CIS-Fs in figures) and cis-males (PRE CIS-Ms in figures) (where levels of endogenous sex hormones are very low), the proportions of CD19^+^CD27^+^IgD^−^ class-switched memory B cells and of IgG^+^ class-switched memory B cells were not significantly different between prepubertal cis-females and prepubertal cis-males (Fig. 3, A and B; and Fig. S1, E–H). Furthermore, when each prepubertal sex was compared with their postpubertal sex-matched counterparts, significantly increased proportions of total class-switched memory B cells were observed in postpubertal versus prepubertal cis-females (Fig. 3, C and D). Finally, using a smaller, independent validation cohort, which provided access to an older age range of individuals, we were able to recapitulate our findings that cis-females have a higher frequency of class-switched memory B cells than cis-males in whole blood samples from individuals aged 20–40 years and also additionally identified that there is no sex difference in the frequency of CD19^+^CD27^+^IgD^−^ class-switched memory B cells between older cis-males (60+ years) and age-matched postmenopausal cis-females (Fig. 3, E–H). This is due to a decrease in the levels of CD19^+^CD27^+^IgD^−^ class-switched memory B cells in postmenopausal cis-females compared with younger 20–40-year-old cis-females (Fig. 3, I and J). Importantly, the percentage of CD19^+^CD27^+^IgD^−^ class-switched memory B cells did not correlate with the age of donors of either sex in both our initial analysis and our validation analysis, suggesting that the rise in hormone levels at puberty may be of greater significance than the age-related increase in the cumulative exposure to antigens (Fig. S1, I–L). Collectively, these results suggest that while sex chromosome–encoded genes play a potentially dominant role in the regulation of the B cell transcriptome, the presence of female sex hormones is regulating the frequency of CD19^+^CD27^+^IgD^−^ class-switched memory B cells.

### Estrogen has the capacity to directly modulate the expression of key transcriptional regulators of CSR

To understand whether estrogen had the capacity to directly influence human B cell biology, we next assessed the expression of sex hormone receptors (*ESR1,* estrogen receptor α; *ESR2,* estrogen receptor β; and *AR*) in total B cells isolated from postpubertal healthy cis-females and cis-males (Fig. 3, K and L). In both sexes, both *ESR1* and *ESR2* were expressed in B cells (validating the findings of Phiel et al. [2005] that ERβ is the dominant sex hormone receptor on human B cells), with minimal expression of *AR*. To assess the potential ability of estrogen to directly regulate CSR, a set of genes known to regulate B cell class switching (Table S2) were assessed for putative transcriptional factor (TF)–binding sites by comparing this class-switching gene set to ChIP-X Enrichment Analysis 3 (ChEA3) libraries of TF target gene sets. Of the 1,632 TFs included, *ESR2* was the second highest ranked TF (Fig. 3 M), demonstrating a direct ability for *ESR2*, but not *ESR1*, to regulate the expression of these genes. RNAseq gene counts of three class-switching genes (*AICDA*, *HOXC4*, and *RNF8*) also show a strong trend toward an increase in cis-female compared with cis-male B cells (Fig. 3, N–P; Table S2 lists P values for count comparisons of the other genes in this set, as well as their main function[s]). Interestingly, studies from murine B cells have also demonstrated that B cells express estrogen receptors and that estrogen can regulate expression of *AICDA/Aicda* via *HOXC4/HoxC4* (Mai et al., 2010). Future mechanistic studies are needed to understand whether estrogen does indeed directly regulate class switching in humans similarly to mice or whether alternative mechanisms are at play.

### The impact of endogenous estrogens on class-switched memory B cell frequency is dependent on sex chromosomal complement

**Figure S2.**
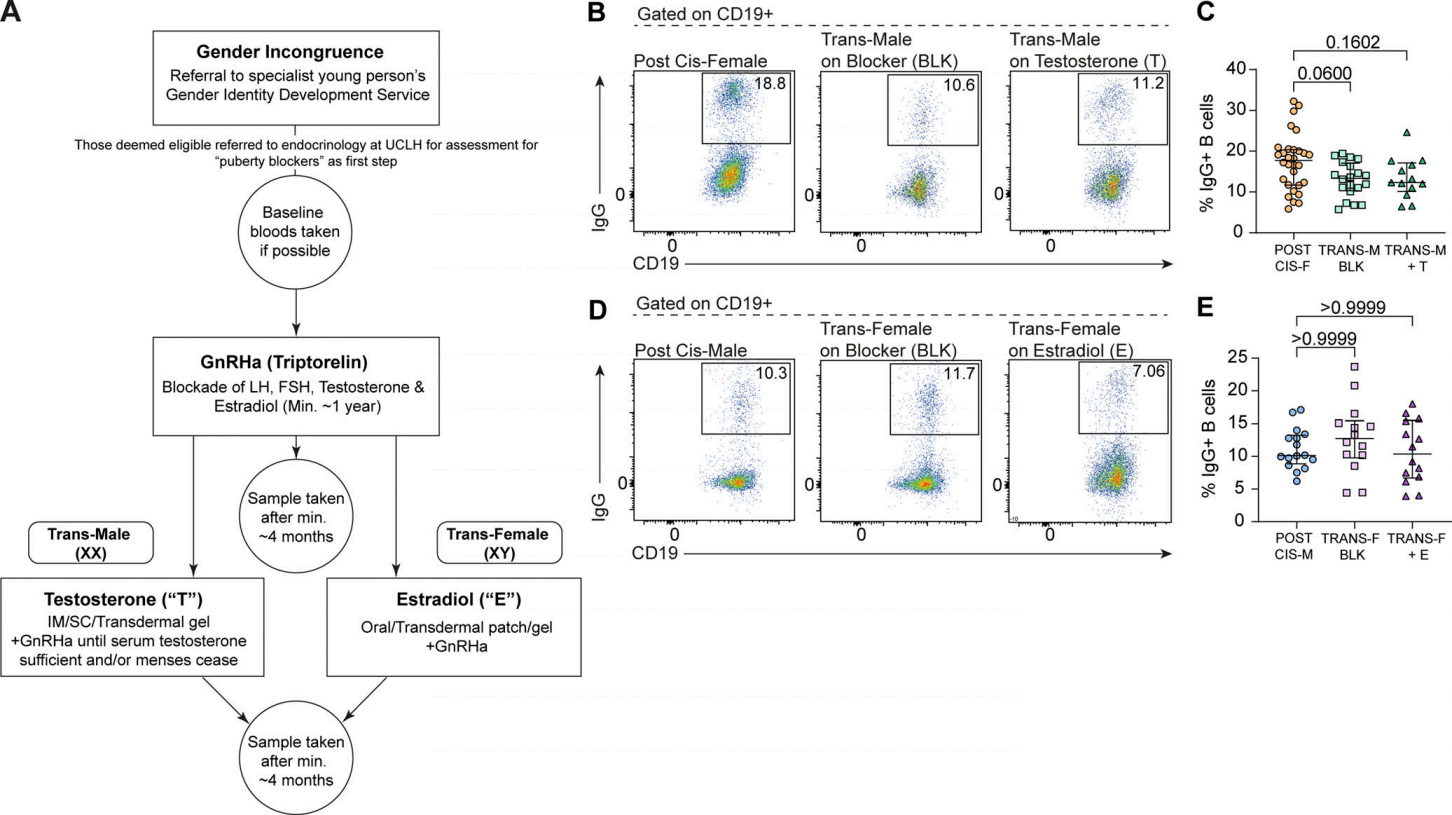
**The frequency of IgG** ^**+**^ **B cells are influenced by sex hormonal modulation in transgender people. (A)** Summary of gender identity clinic treatment pathway and sample schedule. Treatment is prescribed on a case-by-case basis, based on individual country guidelines. This flowchart outlines the most commonly pursued routes by young persons referred to England’s National Health Service (NHS) GIDS. Summarized from guidelines outlined in Hembree et al. (2017). **(B and C)** Representative flow cytometry plots (B) and scatter plot (C) comparing levels of IgG^+^ B cells between postpubertal cis-females (POST CIS-F; *n* = 30), trans-males on puberty blockers (TRANS-M +BLK; *n* = 20; P = 0.0600), and trans-males on testosterone (TRANS-M +T; *n* = 13; P = 0.1602). **(D and E)** Representative flow cytometry plots (D) and scatter plot (E) comparing levels of IgG^+^ B cells between postpubertal cis-males (POST CIS-M; *n* = 17), trans-females on puberty blockers (TRANS-F +BLK; *n* = 14; P ≥ 0.9999), and trans-females on estradiol (TRANS-F +E; *n* = 14; P ≥ 0.9999). Note: Representative plots shown in B for postpubertal cis-females and in D for postpubertal cis-males are duplicated from Fig. 1 G for ease of comparison with their respective transgender cohorts. Kruskal–Wallis test with Dunn’s post hoc test and median + IQR shown (C and E). LH, luteinizing hormone; FSH, follicle-stimulating hormone; IM, intramuscular; SC, subcutaneous.

Together, our data show that both sex hormones and sex chromosomes play a pivotal role in influencing the heightened frequency of CD19^+^CD27^+^IgD^−^ class-switched memory B cells observed in postpubertal and pre-menopausal cis-females compared with age-matched males but do not directly address the relative contributions of both. Thus, we next utilized a unique, gender-inclusive cohort that, due to modulation of sex hormone availability on both XX and XY chromosomal backgrounds, provides a novel opportunity to separate these contributing elements in vivo. Full explanations of the GAHT pathways are detailed in the Materials and methods section and Fig. S2 A. Briefly, both trans-males (XX karyotype, registered female at birth) and trans-females (XY karyotype, registered male at birth) were commenced on “puberty blocker” treatment, suppressing estrogen/P4 and testosterone production. Some trans-males then went on to take gender-affirming testosterone treatment, while some trans-females went on to take gender-affirming estradiol (no additional P4 or anti-androgen medication with progestogenic characteristics was administered).

**Figure 4.**
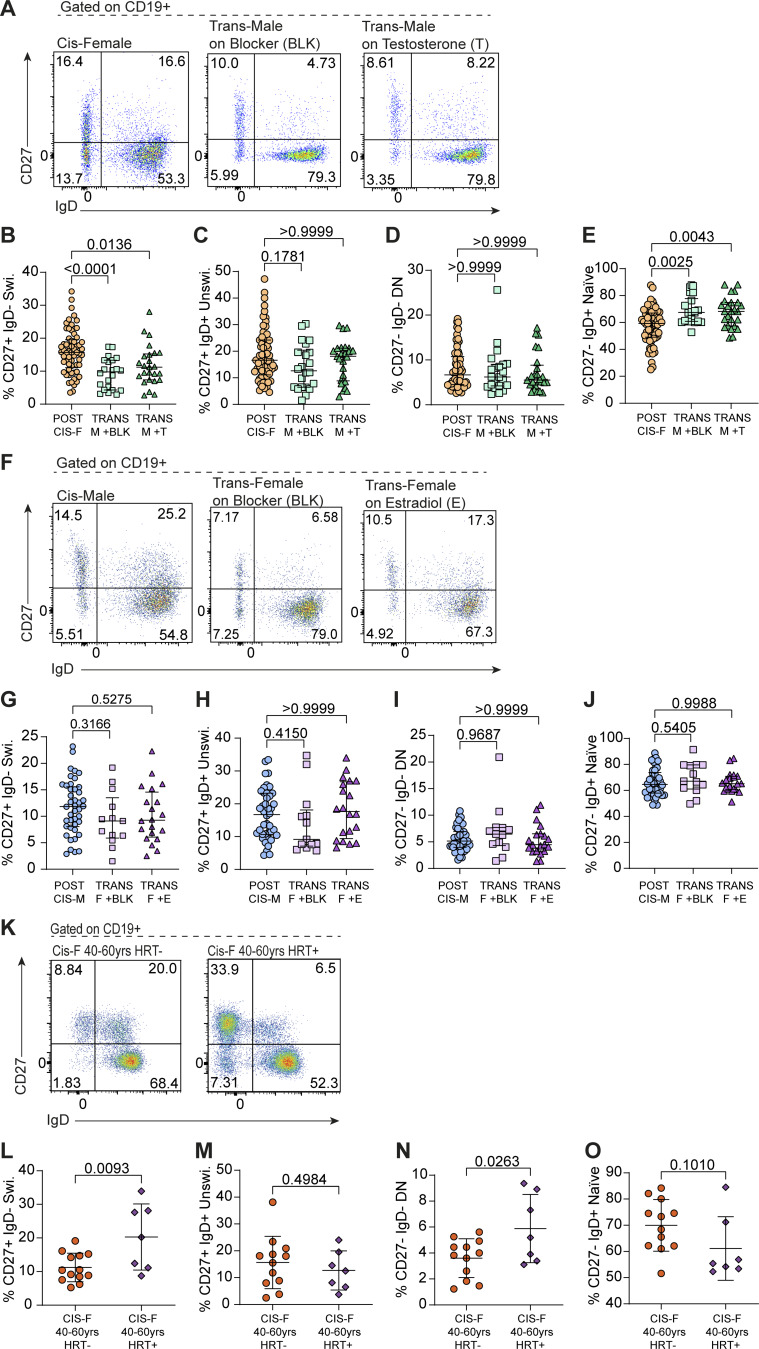
**Differential impact of hormones on the frequency of class-switched memory B cells are dependent upon sex chromosomal complement. (A–E)** Representative flow cytometry plots and scatter plots showing relative percentages of (B) class-switched memory B cells (Swi.; CD27^+^ IgD^−^), (C) unswitched (Unswi.; CD27^+^ IgD^+^), (D) DN (CD27^−^ IgD^−^), and (E) naïve (CD27^−^ IgD^+^) B cells in postpuberal cis-females (POST CIS-F; *n* = 61) compared with trans-males on GnRHa puberty hormone blockers (TRANS-M +BLK; *n* = 23; Swi. P = <0.0001; Unswi. P = 0.1781; DN P ≥ 0.9999; and naïve P = 0.0025) ± gender-affirming testosterone treatment (TRANS-M +T; *n* = 24; Swi. P = 0.0136; Unswi. P ≥ 0.9999; DN P ≥ 0.9999; and naïve P = 0.0043). **(F–J)** Representative flow cytometry plots and scatter plots showing relative percentages of (G) class-switched memory B cells (Swi., CD27^+^ IgD^−^), (H) unswitched (Unswi.; CD27^+^ IgD^+^), (I) DN (CD27^−^ IgD^−^), and (J) naïve (CD27^−^ IgD^+^) B cells in postpubertal cis-males (POST CIS-M; *n* = 42) compared with trans-females on puberty hormone blockers (TRANS-F +BLK; *n* = 15; Swi. P = 0.3166; Unswi. P = 0.4150; DN P = 0.9687; Naïve P = 0.5405) ± gender-affirming estradiol treatment (TRANS-F +E; *n* = 21; Swi. P = 0.5275; Unswi. P ≥ 0.9999; DN P ≥ 0.9999; and naïve P = 0.9988). **(K–O)** Representative flow cytometry plots and scatter plots showing relative proportions of (L) class-switched memory B cells (Swi., CD27^+^ IgD^−^; P = 0.0093) (M) unswitched (CD27^+^ IgD^+^; P = 0.4984), (N) DN (CD27^−^ IgD^−^; P = 0.0263), and (O) naïve (CD27^−^ IgD^+^; P = 0.1010) B cells in whole blood samples from cis-females aged 40–60 years (CIS-F 40–60 years) who were either not taking HRT (HRT^−^; *n* = 12) or were taking HRT (HRT^+^; *n* = 7). Ordinary one-way ANOVA with Tukey’s test (B, G, and J) or unpaired *t* test (L–O) both with mean + SD, or Kruskal–Wallis test with Dunn’s post hoc test and median + IQR shown (C–E, H, and I), as appropriate to distribution. Following correction for multiple testing, adjusted significance determined as P < 0.01.

We first compared the frequency of CD19^+^CD27^+^IgD^−^ class-switched memory B cells and other B cell subsets between postpubertal cis-females and their karyotype counterparts, trans-males who had received either puberty blockers alone (which blocks the production of natal estrogen/P4; referred to as TRANS-M +BLK in figures) or puberty blockers with additional gender-affirming testosterone (referred to as TRANS-M +T in figures). This analysis demonstrated that trans-males have a reduced frequency of CD19^+^CD27^+^IgD^−^ class-switched memory B cells and IgG^+^ B cells when compared with cis-females ex vivo (Fig. 4, A and B; and Fig. S2, B and C). Importantly, this decrease was observed following the blockade of estrogen/P4 using puberty blocker treatment, with gender-affirming testosterone treatment having no further effect on the frequency of CD19^+^CD27^+^IgD^−^ class-switched memory B cells (Fig. 4, A and B). Assessment of the relative proportions of other B cell subsets demonstrated that unswitched and DN B cells did not differ with gender-affirming treatment, but proportions of naïve B cells were increased in both trans-males on puberty blockers alone and on additional testosterone compared with cis-females (Fig. 4, A and C–E). Conversely, when assessing the levels of class-switched memory B cells in trans-females in receipt of puberty blockers (which block production of natal testosterone; TRANS-F +BLK in figures) or additional gender-affirming estradiol treatment (referred to as TRANS-F +E in figures) compared with postpubertal cis-males, we found that there was no difference in both CD19^+^CD27^+^IgD^−^, IgG^+^ class-switched memory B cell populations or any other B cell subset when compared with their chromosomal counterparts, cis-males (Fig. 4, F–J; and Fig. S2, D and E). Finally, to further interrogate whether this may represent a difference between endogenous versus exogenous estrogen and/or an effect that was specific to the XX chromosomal background, we examined the impact of HRT on B cell phenotype in cisgender women of menopausal age. Those taking HRT had significantly higher frequencies of CD19^+^CD27^+^IgD^−^ class-switched memory B cells than those who were not (Fig. 4, K and L). Interestingly, while not impacting the levels of naïve or unswitched B cells, HRT was also associated with an increased frequency of DN B cells, albeit to a lesser extent than CD19^+^CD27^+^IgD^−^ class-switched memory B cells (Fig. 4, K and M–O). Collectively, these results demonstrate that estrogen is associated with increased levels of CD19^+^CD27^+^IgD^−^ class-switched memory B cells ex vivo exclusively in individuals with an XX chromosomal background. This finding is of pertinence as there is a paucity of good-quality literature on transgender health and long-term outcomes for transgender populations beyond a few notable recent papers (Lakshmikanth et al., 2024; Grünhagel et al., 2023) and demonstrates that hormonal transition may lead to distinct immunobiological gender phenotypes. It is also important as little is known regarding how hormone therapy impacts the immune system in postmenopausal cis-females and how this may influence disease outcomes. These observations may be of growing importance as we approach the era of precision medicine.

Our study represents the first in-depth study of B cell immunobiology in cisgender and transgender individuals, providing a unique human-only in vivo insight into the signals regulating the frequency of class-switched memory B cells. Due to the pathways by which these individuals are recruited, there are some limitations to our study. As postpubertal cisgender people were not recruited through a clinical pathway, we are unable to obtain total lymphocyte counts from these donors, meaning our data are based on cellular frequency rather than absolute numbers. Further, due to the length of time that would be required to obtain both pre and postpubertal matched samples from the same cisgender participants, these two cohorts do not represent a longitudinal analysis. B cell class switching is subjected to many in vivo and in vitro environmental factors, which are challenging to control for in this model. In addition, trans-females do not receive P4 or additional specific anti-androgen treatment as part of gender-affirming care (Milionis et al., 2022), and our study does not rule out a possible additional role for physiological P4 in the regulation of B cell class switching, which has been suggested by limited murine literature (Pauklin and Petersen-Mahrt, 2009; Wong et al., 2015; Hughes and Choubey, 2014). However, the P4 receptor was ranked only 321st out of the 1,632 TFs assessed for CSR gene-binding sites, suggesting that estrogens may be the dominant hormones influencing this process specifically. In this paper, we do not show direct mechanistic evidence for estrogen’s ability to regulate class switching, and it is important to consider alternative hypotheses. For example, it has been shown that IgG^+^ B cells are positively selected over IgM^+^ B cells in germinal centers (Sundling et al., 2021), which can lead to a higher proportion of IgG^+^ B cells amongst postgerminal center memory populations that is independent of the CSR process. In addition, differences in the expression of *HRK* (which encodes a pro-apoptotic protein) between cis-males and cis-females may lead to altered survival of different B cell subpopulations and therefore warrant further study. This said, in mice, estrogen has been demonstrated to upregulate *HoxC4* (Mai et al., 2010; Pauklin et al., 2009), a TF that binds directly to the *Aicda* gene promoter, potentiating CSR. Estrogen-response elements have also been described within the immunoglobulin heavy chain locus (Jones et al., 2016), where estrogen:ERα binding facilitates CSR by preventing ERα from binding and blocking enhancers that support DNA looping/remodelling (Jones et al., 2019). Interestingly, DN B cells, which are also class-switched, did not exhibit the same strong gender differences as canonical CD19^+^CD27^+^IgD^−^ class-switched memory B cells, suggesting that different B cell activation pathways may be differentially regulated by sex hormones and/or chromosomes. Although we did find a small difference in the levels of DN B cells in postpubertal cis-females compared with cis-males, the impact of estradiol on this population seemed to be more complex, as although HRT was associated with a higher level of DN B cell in postmenopausal women, neither blockade of estrogen in trans-males nor supplementation of estradiol in trans-females impacted this cell type when compared with karyotype-matched cisgender people. Importantly, there is evidence that DN B cells, which are enriched for extrafollicularly or atypically differentiated B cells (ABCs), are influenced by the XCI escapee gene *XIST* (the long coding RNA that establishes XCI). *XIST* is dysregulated in SLE and severe COVID-19 (both conditions with a strong sex bias), which leads to an increase in ABCs (Yu et al., 2021; Dou et al., 2024), and perturbation of *Xist* in mice leads to reactivation of *Tlr7*, expansion of ABCs, and spontaneous development of lupus (Huret et al., 2024). In addition, DN B cells/ABCs are well-known to be expanded in response to TLR7 activation, which is X chromosomally encoded (Brown et al., 2022). In our study of how sex determinants affect frequencies of class-switched memory B cells and DN B cells, we also cannot discount the possibility that estrogen influences the expression of XCI escapee genes, and that this impacts our observations. Future studies are warranted to explore the differential roles of sex hormones and sex chromosomes on different B cell activation pathways and the direct mechanisms underpinning the observed differences in the frequency of B cell subpopulations.

Despite these limitations, in this study we demonstrate an important complementary role for sex hormones and sex chromosomes in regulating the frequency of CD19^+^CD27^+^IgD^−^ class-switched memory B cells. Our key observations include that only postpubertal, but not prepubertal or postmenopausal, cis-females have higher frequencies of class-switched memory B cells compared with cis-males. This shows that while sex chromosomes drive most of the sex differences in B cell transcriptomes, this is insufficient to alter the frequency of class-switched memory B cells. Further analysis demonstrates that estrogen may have a potentially direct role in regulating B cell class switching as B cells express high levels of *ESR2*, and genes regulating B cell class switched are enriched for *ESR2*-binding sites. Using a unique cohort of transgender individuals and postmenopausal cis-females receiving hormone therapy, we finally demonstrate that estrogen works in tandem with an XX chromosomal background to increase the frequency of CD19^+^CD27^+^IgD^−^ class-switched memory B cells. Blockade of estrogen on an XX chromosomal background reduces the frequency of class-switched memory B cells, whilst addition of estrogen only impacted the levels of CD19^+^CD27^+^IgD^−^ class-switched memory B cells in individuals with an XX chromosomal background. Thus, sex hormones differentially regulate the frequency of CD19^+^CD27^+^IgD^−^ class-switched memory B cells in the presence of different sex chromosomes, suggesting that cisgender and transgender individuals and individuals receiving hormone therapy in general have distinct immune phenotypes. This study adds to the growing evidence that sex and gender are critical factors to be considered in immunological studies. It also demonstrates that improvements in diversity and inclusion practices within medical research will not only advance our scientific understanding of sex biases in disease outcomes but also potentially shed light on novel strategies for personally tailored healthcare.

## Materials and methods

### Human participants—Peripheral blood mononuclear cell (PBMC) cohort

Full demographic information for participants is shown in Table 1. Postpubertal cisgender healthy controls were recruited from the community, while prepubertal cisgender controls were recruited from otherwise healthy children undergoing elective corrective dental or urological surgeries at University College London Hospital (UCLH). “Postpuberty” was self-reported by participants using a visual questionnaire as “Tanner stage 4–5,” and “prepuberty” was parentally reported as “Tanner stage 1.” Participants were screened for any history of autoimmune disease, relevant endocrinological conditions, serious or current infections, and vaccination within the 3 wk prior to blood donation.

Trangender healthy controls were recruited from the young persons’ Gender Identity Development Service (GIDS) at UCLH, between 2016 and 2020. Healthy, young transgender people were recruited to donate peripheral blood samples at determined intervals of GAHT regimens. Briefly, both trans-males (XX karyotype, registered female at birth) and trans-females (XY karyotype, registered male at birth) are commenced on gonadotrophin-releasing hormone analogues (GnRHa), commonly referred to as “puberty blockers.” Overstimulation of the GnRH receptor leads to the eventual cessation of both estrogen and subsequently P4 and testosterone production. After a minimum of 12+ mo on GnRHa alone, those seeking further virilization or feminization and deemed Gillick competent (in the UK, persons under 16 years can consent to their own medical treatment if they are felt to have suitable intelligence, competence, and understanding to fully appreciate what is involved in the treatment [Griffith, 2016]) may receive additional testosterone (trans-males) or estradiol (trans-females), respectively, using gradually increasing dosages designed to mimic natal puberty. Samples are obtained when the young people have been on blockers (alone) for a minimum of 6 mo and then again when they have been on additional GAHT for a further minimum of 4 mo. Blocker treatment is maintained until natal estrogen/testosterone levels are sufficiently suppressed by GAHT (and/or later gonadectomy in adulthood). This treatment pathway is also outlined in Fig. S2 A.

Of the trans-female participants, all were in receipt of GnRHa “puberty blocker” treatment for at least 6 mo, and 20 had been additionally taking gender-affirming estradiol (oral tablet or transdermal patch) for a further 4.5 mo or more. Of the trans-male participants, all were also in receipt of at least 6 mo of GnRHa blocker treatment, with 25 on additional gender-affirming testosterone (intramuscular injection or transdermal gel) for a further 4 mo or more. Seven trans-males had acquired sufficient testosterone trough levels to dispense with GnRHa (required only until testosterone levels alone will sufficiently suppress natal estrogen). All individuals in receipt of GnRHa blocker ± gender-affirming hormones are encouraged to take calcium and vitamin D supplementation. 78/80 participants were deemed to have completed pubertal development as their birth-registered sex (Tanner stage 4–5; based on both self-reported and clinician-confirmed Tanner stage questionnaires) prior to the commencement of any treatment, and 2/80 (both trans-females) were assessed as late Tanner stage 3.

Informed consent was taken from all volunteers aged 16 years or over. For those aged 6–15, or any person deemed not to have capacity; parental/guardian consent was taken (alongside assent from the volunteer themselves, where possible). This study received ethical approval from Health Research Authority, ethical approval reference: REC11/LO/0330 (London-Harrow Research Ethics Committee); samples and data were shared in accordance with the guidelines set by the Center for Adolescent Rheumatology Versus Arthritis at UCL, UCLH, and GOSH. Basic health, demographic, and puberty Tanner staging questionnaires were completed by/for all participants, and anonymized data were stored in compliance with data protection laws.

Venous whole blood was collected in heparin-coated vacutainers. In those under 18 years, this was 2 ml of blood per kg of weight, not exceeding a total of 20 ml per sample. In those aged 18+, 20–60 ml was taken.

### PBMC isolation and serum processing

Within 2 h of sample collection, blood was diluted 1:1 with Roswell Park Memorial Institute medium (RPMI)-1640 containing L-glutamine and sodium bicarbonate (Sigma-Aldrich) supplemented with 100 IU/μg/ml penicillin/streptomycin (Sigma-Aldrich) and 10% heat-inactivated FBS (Gibco). SepMate tubes (Stemcell) were layered with Ficoll-Paque Plus (GE Healthcare), followed by the diluted sample, and then centrifuged at 1,200 *g* for 10 min. After further centrifugation of the top layer, pelleted isolated PBMCs were resuspended and counted using a hemocytometer before being frozen in freezing media consisting of FBS with 10% DMSO (Sigma-Aldrich). Nalgene “Mr Frosty” containers were used for controlled freezing at −80°C, and samples were then transferred to liquid nitrogen storage.

### Human subjects—Whole blood cohort

Ethics were approved by the Queen Mary Ethics of Research Committee reference number QMERC23.059. Individuals 18 years or older were recruited into the study, and full written informed consent was obtained from each donor, and up to 40 ml of peripheral blood was taken from each donor and processed as described below. Individuals were excluded from the study if they had a recent history (≤5 years) of neoplasia, immunosuppressive disorders that required immunosuppressive medication, were anemic, or had a current pregnancy.

### Flow cytometry—PBMC cohort

PBMC samples were thawed and washed once in warm RPMI-1640 media with 10% FBS (“cRPMI”; complete RPMI). Cells were counted with a hemocytometer. Thawed cells were plated at either 250,000, 500,000, or 1 million cells per well (depending on the panel) in a 96-well plate and centrifuged at 500 *g* for 5 min to pellet. Following resuspension in PBS, they were stained with fixable blue dead cell stain (1:500) (L23105; Thermo Fisher Scientific) for 15 min at room temperature in foil. After washing, cells receiving the immunoglobulin panel stain (CD19-BV510 [HIB19], CD27-BV711 [O323], IgD-BV421 [IA6-2], IgM-FITC [MHM-88], IgA-APC [IS11 8E10], IgE-PE [IGE21], and IgG-BUV737 [G18–145]) were first resuspended in FACS buffer (1× Dulbecco’s PBS [DPBS] without calcium or magnesium [SH30028.02; GE Healthcare Hyclone] + 5 ml FBS + 2 mM EDTA) with Human TruStain FcX Fc Receptor Blocking Solution (1:20; BioLegend) for 15 min at room temperature in foil. Without washing, cells were spun down to pellet. For the surface staining, cells were incubated in foil for 30 min at room temperature with the relevant antibody panel (Panel 1—B cells and monocytes: BADCA2-PE [201A], CD56-FITC [5.1H11], CD16-APC [3G8], CD123-PE/Cy7 [6H6], HLA-DR-PERCPCy5.5 [L243], CD24-APC/Cy7 [ML5], CD14-AF700 [63D3], IgD-BV421 [IA6-2], CD19-BV510 [HIB19], CD38-BV605 [HIT2], CD11c-BV785 [3.9], CD27-BV711 [0323], CD3-FITC [HIT3a], and CD66b-FITC [G10F5]; panel 2—T cells and natural killer cells: CCR7-PE [G043H7], TCRγδ-FITC [11F2], CD25-APC [BC96], CD56-PE/Cy7 [MEM-188], CD45RO-PERCPCy5.5 [UCHL1], CD4-APC/Cy7 [OKT4], CD8-AF700 [SK1], CXCR3-BV421 [G025H7], CCR6-BV510 [G034E3], CD3-BV605 [OKT3], CXCR5-BV785 [J252D4], CD127-BV711 [A019D5], and CD45RA-PEDAZZLE [HI100]), diluted in FACS buffer. After washing, surface stain-only panels were fixed with 2% paraformaldehyde fixative for 15 min in foil at room temperature before washing and resuspension in FACS buffer and storage at 4°C. Panels requiring intracellular staining were fixed using the eBioscience Foxp3 staining kit (00–5523-00) Fix/Perm for 15 min in the dark, before washing and staining with the relevant intracellular antibody mix(es) for 40 min in the dark, followed by washing with perm buffer and resuspension in FACS buffer for 4°C storage.

All samples were run the following day on a BD LSRII flow cytometer, with as many cellular events as possible recorded per sample. Data were analyzed using Flowjo v10, with a minimum of 100 events in the parent gate of any population used in analysis. Gating strategies were determined using Fluorescence Minus Ones.

### Flow cytometry—Whole blood cohort

Whole blood was stained with the following cell surface antibodies: CD16-BV785 (3G8) and IgD-Pacific Blue (IA6-2) from BioLegend and CD27-BUV395 (L128), CD19-BUV496 (SJ25C1), and CD3-BUV805 (UCHT1) from BD Biosciences and Live/Dead Blue from Thermo Fisher Scientific. Cells were incubated with antibodies for 45 min at 4°C. After incubation red blood cells were removed using FACs Lysis Buffer (BD Biosciences). Samples were subsequently washed twice in PBS and then assessed by flow cytometric analysis on a Cytek Aurora. Data were analyzed using FlowJo Version X (BD Biosciences).

### FACS

Samples were thawed and counted as above, before staining with Zombie NIR Fixable dye (BioLegend) in cold DPBS on ice for 20 min. After washing, samples were stained with the following antibody panel: CD4-BUV395 (SK3), CD8-BV785 (RPA-T8), CD19-AF488 (HIB19), and CD14-PE/Cy7 (M5E2), in cold FACS buffer, on ice for 20 min. They were then washed and filtered into filter-capped FACS tubes in cold FACS buffer and sorted into cold DPBS using a BD FACSAria flow cytometry cell sorter.

### RNA extraction

RNA was extracted and isolated from sorted CD4/CD8/CD14/CD19^+^ cells and from MCF7 cells as a control, known to be positive for hormone receptors (Horwitz et al., 1975). The PicoPure RNA Isolation Kit (Thermo Fisher Scientific) was used, following the manufacturer’s guidelines. The RNA columns were additionally incubated with RNase-free DNase (Qiagen) at room temperature for 15 min during this process. Eluted RNA was quantified using a NanoDrop spectrophotometer.

### RT-PCR for cDNA synthesis

cDNA was synthesized from RNA following the manufacturer’s instructions of the qScript cDNA SuperMix (Quantabio). The GeneAmp PCR System 9700 Thermocycler was used with the following settings: 5 min at 25°C, 30 min at 42°C, 5 min at 85°C, and hold at 4°C.

### Quantitative PCR (qPCR)

Applied Biosystems TaqMan Fast Advanced Master Mix (Thermo Fisher Scientific) was added to cDNA for fluorescence quantification on an AriaMx Real-time qPCR System (Agilent Technologies) using the following thermal profile: UDG (DNA) 2 min 50°C, polymerase activation hold 95°C 2 min, denature 95°C 1 s, anneal/extend 60°C 20 s.

TaqMan probes for *AR* (Hs00171172_m1, 4331182), ERα (“*ESR1*”—Hs00174860_m1, 4331182), ERβ (“*ESR2*”—Hs00230957_m1, 4331182), *AICDA* (“*AICDA*”—Hs00757808_m1, 4331182), and housekeeping gene *RPLP0* (60S acidic ribosomal protein P0) (4333761T) were purchased from Thermo Fisher Scientific and used at the manufacturer’s recommended concentrations for each reaction. Agilent Aria 1.7 software was used to prepare the data, and Microsoft Excel 2008 was used for analysis. Data were normalized to the reference gene *RPLP0*. The delta-Ct (Livak and Schmittgen, 2001) method of quantification was used to ascertain relative gene expression of the target genes.

### RNAseq

RNAseq was performed by UCL Genomics. Briefly, libraries were prepped with either the KAPA mRNA HyperPrep Kit or the TruSeq Stranded mRNA Library prep. Paired-reads mapping was performed using STAR aligner (Dobin et al., 2013) (Ref genome: GRCh38), and aligned reads were summarized with featureCounts. Quality control was conducted on the bulk RNAseq reads count table obtained from featureCounts to ensure data quality. Samples with <5 million reads were excluded, as these were deemed insufficient for reliable analysis.

Principal component (PC) analysis was subsequently employed to identify outliers within each cell population. It was assumed that samples from the same cell population would cluster together. PC analysis was performed on the filtered count table, and the first two PCs (PC1 and PC2) were plotted to visualize sample clustering. For each cell population, the center and SDs of the clusters were calculated. Samples deviating >4 SDs from the cluster center were identified as outliers and removed. This process ensured that only high-quality, reliable samples were retained for downstream analysis. Detailed RNAseq data analysis workflow describing quality control, alignment, and generation of raw transcript counts can be found in the following GitHub repository: https://github.com/WedderburnLab/RNAseq-Pipeline. Read counts were kit corrected with CombatSeq (Zhang et al., 2020) prior to differential gene expression analysis.

Statistical analysis and visualization of transcriptional data were performed using R software and Bioconductor packages (Gentleman et al., 2004), including DESeq2 (Anders and Huber, 2010; Love et al., 2014) and EnhancedVolcano (Blighe et al., 2022).

Genes for which ≥50% of the control group had counts of 10 or less were excluded from the analysis. DESeq2 in R was used to carry out DEG analysis between two selected groups to calculate the fold changes and the adjusted P value (Padj) for significance of differences (adjusted for multiple testing using Benjamini–Hochberg’s false discovery rate method). Both age of donor and batch that the sample was sent in were controlled for within the analysis model. A Padj cut-off of <0.05 was applied using Microsoft Excel to generate DEG lists for each comparison. Those with a positive fold change were considered to be upregulated, and those with a negative fold change to be downregulated. Detailed RNAseq data analysis workflow describing quality control, alignment, and generation of raw transcript counts can be found in the following GitHub repository: https://github.com/WedderburnLab/RNAseq-Pipeline.

Pathway enrichment analysis was conducted with separate lists of up- and downregulated genes for each comparison, using Metascape (Zhou et al., 2019) to reveal significantly up/downregulated “GO: BP” pathways (Aleksander et al., 2023; Ashburner et al., 2000) within each comparison. Pathways were ranked by P value into bar charts, shown alongside the “enrichment score” (the ratio of the proportion of genes in the list that were associated with the enriched term to the proportion of genes in the genome that are associated with the enriched term). For reduction of redundancy, the program clusters similar enrichment terms, with the most significant term of each cluster represented on the bar chart. DEG lists were further summarized in heatmaps (created with Clustvis [Metsalu and Vilo, 2015], using Euclidean distance and the average method for clustering of rows) for each comparison, demonstrating the clustering of each group by expression of the listed genes. Targeted gene analysis using the gene set detailed in Table S2 was carried out using count data (transcripts per million) for each sample, and analyzed using GraphPad Prism. Briefly, the lists of genes from the “GO Biological Pathways” ontology pathway “GO:0045190- Isotype Switching,” alongside the lists from “GO:0045191- regulation of isotype switching” and “GO:0048291- regulation of isotype switching to IgG” pathways were cross-examined, and duplicates were removed. Also removed were any genes deemed not to be expressed on human B cells, according to The Human Protein Atlas (Uhlen et al., 2019), and the accompanying supporting literature from the GO website (Aleksander et al., 2023; Ashburner et al., 2000). Genes *HOXC4* and *BACH2* were included, given their prominence in the literature on sex differences in CSR to date. To assess enrichment of TF-binding sites in these genes, genes were inputted into the ChEA3 online tool (Keenan et al., 2019).

### Statistical analysis

Statistical analysis was carried out using GraphPad Prism 10. Unpaired *t* tests, Mann–Whitney U tests, one-way ANOVA with Tukey’s test, or Kruskal–Wallis tests with Dunn’s test (dependent upon normality of each dataset and number of groups being compared) were used to determine differences between groups. P values adjusted for multiple testing (in Fig. 1) were calculated by dividing 0.05 by the number of tests performed.

### Figures

Figures and graphical abstract were created using Adobe Illustrator 2025. The graphical abstract used human figure outlines from Servier Medical Art (https://smart.servier.com), licensed under CC 4.0 https://creativecommons.org/licenses/by/4.0/, which were modified as required.

### Online supplemental material

Fig. S1 shows the comparative levels of IgA^+^ and IgE^+^ B cells between postpubertal cis-females versus cis-males, as well as comparison of levels of IgG^+^ B cells between prepubertal cis-females versus cis-males and between prepubertal cis-females and cis-males with their postpubertal gender counterparts. It also includes correlation analysis of age versus proportion of class-switched B cells in cis-females and cis-males. Fig. S2 delineates the treatment pathway for transgender young people in the UK at the time of recruitment to research (2016–2020). It also compares levels of IgG^+^ B cells between cis-females versus trans-males on puberty blocker ± gender-affirming testosterone treatment and between cis-males versus trans-females on puberty blocker ± gender-affirming estradiol treatment. Table S1 provides a list of all DEGs (up- or downregulated) between B cells from postpubertal cis-females versus cis-males. Table S2 describes the set of genes associated with CSR that were used in Fig. 3, L–O, with P values for the comparison of their gene counts in B cells from postpubertal cis-females versus cis-males.

## Supplementary Material

Table S1 shows the significantly up- or downregulated genes in B cells from postpubertal cis-females versus cis-males.

Table S2 shows the class-switching gene set used in Fig. 3, L–O.

## Data Availability

RNAseq data can be found at ArrayExpress repository (accession number: E-MTAB-14599). These data will be available from date of manuscript publication.
