# Supplementary material for: Estrogen influences class-switched memory B cell frequency only in humans with two X chromosomes
Source: J Exp Med. 2025 Mar 6;222(4):e20241253. doi: 10.1084/jem.20241253 (PMC11893172; doi:10.1084/jem.20241253)
Supplement: Table S1 — shows the significantly up- or downregulated genes in B cells from postpubertal cis-females versus cis-males. [file jem_20241253_tables1.docx]

**Table S1. Significantly up- or downregulated genes in B cells from post-pubertal cisgender females (POST CF) vs. post-pubertal cisgender males (POST CM)**

| **Down in POST CF vs. POST CM** | **Chromosome** | **Up in POST CF vs. POST CM** | **Chromosome** |
| --- | --- | --- | --- |
| \| CHIT1 \| \| --- \| \| ZAP70 \| \| PRF1 \| \| AC136475.3 \| \| PXN \| \| HRK \| \| GZMH \| \| GPR68 \| \| CCL5 \| \| AC254562.3 \| \| KDM5D \| \| DDX3Y \| \| ZFY \| \| PRKY \| \| USP9Y \| \| RPS4Y1 \| \| TXLNGY \| \| TTTY14 \| \| UTY \| \| EIF1AY \| \| BCORP1 \| \| AC010086.1 \| \| TTTY10 \| \| LINC00278 \| \| ANOS2P \| \| AC010889.1 \| \| RPS4Y2 \| \| AC244213.1 \| | \| 1 \| \| --- \| \| 2 \| \| 10 \| \| 11 \| \| 12 \| \| 12 \| \| 14 \| \| 14 \| \| 17 \| \| 22 \| \| Y \| \| Y \| \| Y \| \| Y \| \| Y \| \| Y \| \| Y \| \| Y \| \| Y \| \| Y \| \| Y \| \| Y \| \| Y \| \| Y \| \| Y \| \| Y \| \| Y \| \| Y \| | \| EIF1AXP1 \| \| --- \| \| MARCHF7 \| \| RGPD2 \| \| RGPD1 \| \| ZNF141 \| \| BNIP3P41 \| \| TMEM106B \| \| MS4A7 \| \| IRAK3 \| \| EIF2S3B \| \| WDR89 \| \| ZNF681 \| \| ZNF181 \| \| ZNF480 \| \| ZFX \| \| PNPLA4 \| \| SMC1A \| \| TXLNG \| \| USP9X \| \| PUDP \| \| EIF2S3 \| \| KDM6A \| \| ZRSR2 \| \| EIF1AX \| \| PRKX \| \| MAP7D2 \| \| CXorf38 \| \| ERCC6L \| \| TRAPPC2 \| \| RPS4X \| \| DDX3X \| \| INE1 \| \| JPX \| \| XIST \| \| TSIX \| \| BX890604.1 \| | \| 1 \| \| --- \| \| 2 \| \| 2 \| \| 2 \| \| 4 \| \| 4 \| \| 7 \| \| 11 \| \| 12 \| \| 12 \| \| 14 \| \| 19 \| \| 19 \| \| 19 \| \| X \| \| X \| \| X \| \| X \| \| X \| \| X \| \| X \| \| X \| \| X \| \| X \| \| X \| \| X \| \| X \| \| X \| \| X \| \| X \| \| X \| \| X \| \| X \| \| X \| \| X \| \| X \| |
