# Supplementary material for: Estrogen influences class-switched memory B cell frequency only in humans with two X chromosomes
Source: J Exp Med. 2025 Mar 6;222(4):e20241253. doi: 10.1084/jem.20241253 (PMC11893172; doi:10.1084/jem.20241253)
Supplement: Table S2 — shows the class-switching gene set used in Fig. 3, L–O. [file jem_20241253_tables2.docx]

**Table S2. Class-switching gene set used in Fig 3, L–O**

| **Gene** | **Significance to Class-Switch Recombination** | **Cis-F vs. Cis-M gene count**  **p-value** |
| --- | --- | --- |
| *AICDA* | Activation-induced cytidine deaminase. Critical enzyme for the DNA mutations necessary for CSR to occur (Muramatsu *et al.*, 2000). | 0.0825 |
| *ATAD5* | ATPase Family AAA Domain Containing 5. Responsible for unloading proliferating cell nuclear antigen from newly synthesised DNA. In knock-out mice, AID expression, CSR and B cell division are all decreased (Zanotti *et al.*, 2015). | 0.6098 |
| *BATF* | Basic Leucine Zipper ATF-Like Transcription Factor. Controls AID expression (Ise *et al.*, 2011). | 0.2171 |
| *BCL6* | B-cell lymphoma 6. Transcriptional repressor necessary for formation of germinal centres, preventing B cells from becoming short-lived plasma cells (Alinikula *et al.*, 2011). *BCL*-/- mice exhibit increased CSR to IgE and inflammatory symptoms (Harris *et al.*, 1999) as it binds to the E-germline transcription promoter (Audzevich *et al.*, 2013). Depends on ***STAT6*** (Haase *et al.*, 2020)**.** | 0.0705 |
| *BACH2* | A transcriptional regulator which limits B cell differentiation into plasma cells until after AID expression, when CSR and SHM have consequently taken place. Compromised expression in SLE. *Bach2*-/- mice have enhanced extra-follicular CSR & IgG+ autoantibodies (Jang *et al.*, 2019). | 0.9381 |
| *CCR6* | Significant role in switch to IgA and gut immunity (Lin, Ip and Liao, 2017). | 0.1592 |
| *ERCC1* | Excision Repair 1. *ERCC1-XPF* shown to be essential component of DNA repair pathway, interacts with ***MSH2*** in processing or repairing DNA lesions in S regions in CSR (Schrader *et al.*, 2004). | 0.9787 |
| *EXO1* | Exonuclease 1. *Exo*-/- mice had decreased CSR (Bardwell *et al.*, 2004; Eccleston *et al.*, 2011). Acts with ***MSH2*** and ***MLH1*.** | 0.5065 |
| *EXOSC3* | Exosome Component 3. *EXOSC3*-deficient B cells cannot class switch (Pefanis *et al.*, 2014). | 0.1287 |
| *EXOSC6* | Exosome Component 6. Subunit of the exosome, which *may* target *AICDA* deaminase activity toward transcribed dsDNA substrates (Basu et al., 2011). | 0.9777 |
| *HOXC4* | A transcription factor- binds directly to the *AICDA* gene promoter, thus potentiating CSR. Upregulated in SLE & lupus-prone mice. *Hoxc4* -/- mice showed a reduction in IgG2a autoantibodies and IgG kidney deposition (White *et al.*, 2011). Oestrogen has also been demonstrated to upregulate *HoxC4*. In lupus-prone mice, elevated levels of class-switched autoantibodies and chromosomal translocations, induced in a Hoxc4-AID-dependent manner, suggest that oestrogen may play a part in AID dysregulation in SLE (Pauklin *et al.*, 2009; Mai *et al.*, 2010). | 0.0237 |
| *LIG4* | DNA Ligase 4. NHEJ factor (Ghosh and Raghavan, 2021). Mutations in *LIG4* lead to "*LIG4 syndrome*", where patients have less effective or defective V(D)J recombination. Some have a severe immunodeficiency (Altmann and Gennery, 2016). | 0.9957 |
| *MLH1* | MutL Homolog 1. Mismatch repair protein, thought to convert nicks to breaks, acts with ***MSH2* & *EXO1*** (Eccleston *et al.*, 2011). | 0.1574 |
| *MSH2* | MutS Homolog 2. In mice, involved in DNA mismatch recognition. *Msh2*-/- show decreased IgG *in vitro* and *in vivo* (Ehrenstein and Neuberger, 1999). Interacts with ***ERCC1*.** | 0.2826 |
| *MSH6* | MutS Homolog 6. A mismatch repair protein (MMR), its phosphorylation regulates CSR via *DYRK1A* (Stoler-Barak *et al.*, 2023). | 0.1723 |
| *NBN* | Nubrin protein - role in double stranded break repair as part of Mre11/Rad50/Nibrin complex (Piątosa *et al.*, 2012) and recombination of Ig constant genes (Kracker *et al.*, 2005). | 0.0654 |
| *NFKBIZ* | IKBNS-/- B cells in mice have decreased proliferation and CSR to IgG3. Demonstrated role in TLR-medicated T-independent CSR (Touma *et al.*, 2011). | 0.0697 |
| *RNF168* | Ring Finger Protein 168. Double stranded breaks are repaired by *53BP-1*-dependent process - ***RNF8*** & *RNF186* recruit *53BP-1* to site of damage (Ramachandran *et al.*, 2010). | 0.7982 |
| *RNF8* | Ring Finger Protein 8. Key ubiquitination pathway mediator, integral to CSR (Ramachandran *et al.*, 2010). *RNF8* deficient mice show impaired CSR (Li *et al.*, 2010) and *RNF8* knock-out mice had defective CSR and impaired double stranded breaks (Santos *et al.*, 2010). | 0.0141 |
| *STAT6* | Signal Transducer And Activator Of Transcription 6. Crucial role in B cell development & CSR (Wang *et al.*, 2021). In mice, pivotal role in IgG1 and IgE switch (Haase *et al.*, 2020). Linked pathway with ***BCL6*.** | 0.7325 |
| *SWAP70* | Switching B Cell Complex Subunit. SWAP70 knock-out mice exhibit decreased IgE & IgG1 production (Borggrefe *et al.*, 2001) with IgE production controlled via ***STAT6/BCL6.*** | 0.0862 |
| *UNG* | Controversial role in CSR (Yousif *et al.*, 2014). *UNG*-/- mice were deficient in CSR (Rada *et al.*, 2002), unclear mechanism. | 0.1878 |

CSR- class-switch recombination; DNA - deoxyribonucleic acid; ATP- adenosine triphosphate; AID- activation-induced deaminase; SHM- somatic hypermutation; SLE- systemic lupus erythematosus; NHEJ- non-homologous end joining; Ig- immunoglobulin. Mann-Whitney U test used to obtain P-values.
